# Supplementary material for: A tungsten polyoxometalate mediated aqueous redox flow battery with high open-circuit voltage up to 2 V
Source: Nat Commun. 2025 May 19;16:4654. doi: 10.1038/s41467-025-60018-7 (PMC12089354; doi:10.1038/s41467-025-60018-7)
Supplement: Supplementary file 1 — Supplementary Information [file 41467_2025_60018_MOESM1_ESM.pdf]

Supplementary Information

A Tungsten Polyoxometalate Mediated Aqueous Redox Flow Battery with High Open-Circuit Voltage up to 2 V

Weipeng Li[1], Weizhuo Xu[1], Zhaopeng Sun[1], Linning Tang[1], Guohao Xu[1], Xinyue He[1], Yulin Deng[2], Wei Sun[3], Bingjie Zhou[4], Jianfei Song[5], Wei Liu[1]*

[1]School of Chemistry and Chemical Engineering, Central South University, Changsha Hunan 410083, P. R. China

[2]School of Chemical & Biomolecular Engineering and RBI at Georgia Tech, Georgia Institute of Technology, 500 10th Street N.W., Atlanta, GA 30332-0620, USA

[3]School of Chemistry and Chemical Engineering, Shihezi University, Shihezi 832003, PR China

[4]National Engineering Laboratory for Mobile Source Emission Control Technology, China Automotive Technology & Research Center Co., Ltd., Tianjin 300300, P. R. China

[5]Changsha New Energy Innovation Institute, Changsha Hunan 410083, P. R. China

*Correspondence and requests for materials should be addressed to Wei Liu (email: wliu300@csu.edu.cn)

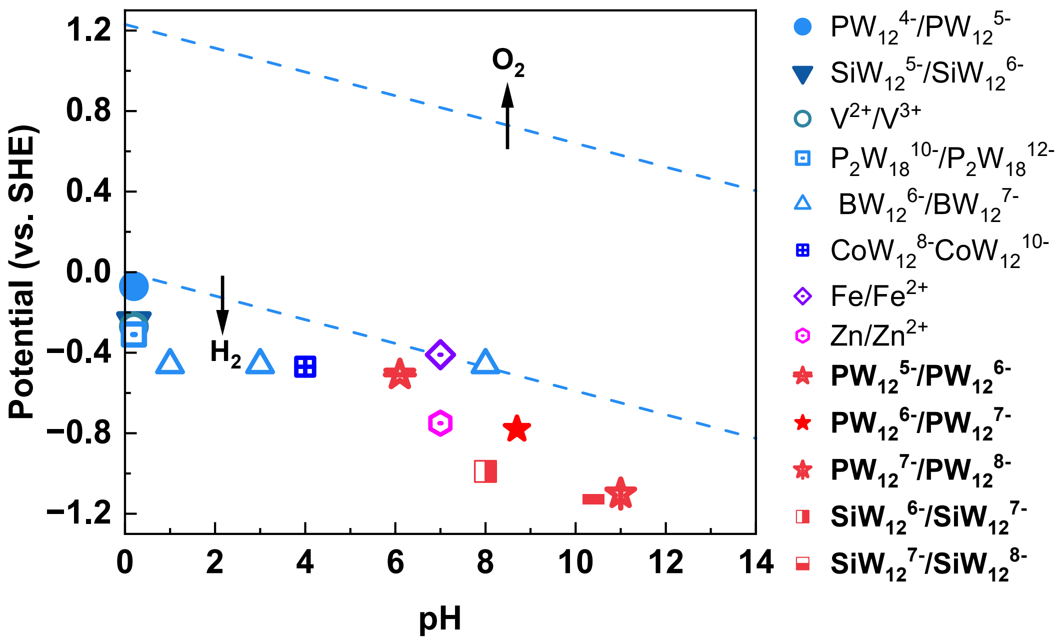

Supplementary Figure 1 | Pourbaix diagram for water splitting potentials at different pH values derived from the Nernst equation and the redox potentials of different redox pairs in this work and reported researches (also see Fig.1c in manuscript).

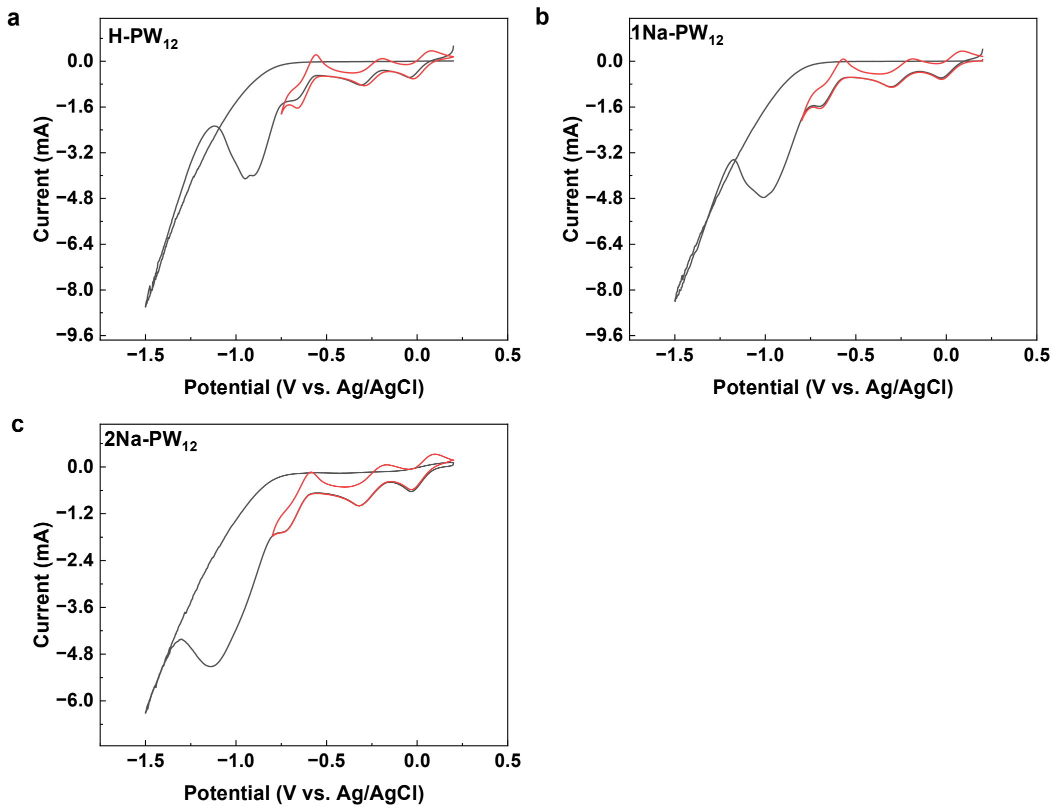

Supplementary Figure 2 | CV measurement. CV curves of H-PW_{12}, 1Na-PW_{12} and 2Na-PW_{12} solutions (0.1 mol l^{-1}) with different test potential windows at a scan rate of 50 mV s^{-1}. GC electrode, Pt electrode and Ag/AgCl (saturated KCl) electrode were used as working electrode, counter electrode and reference electrode respectively.

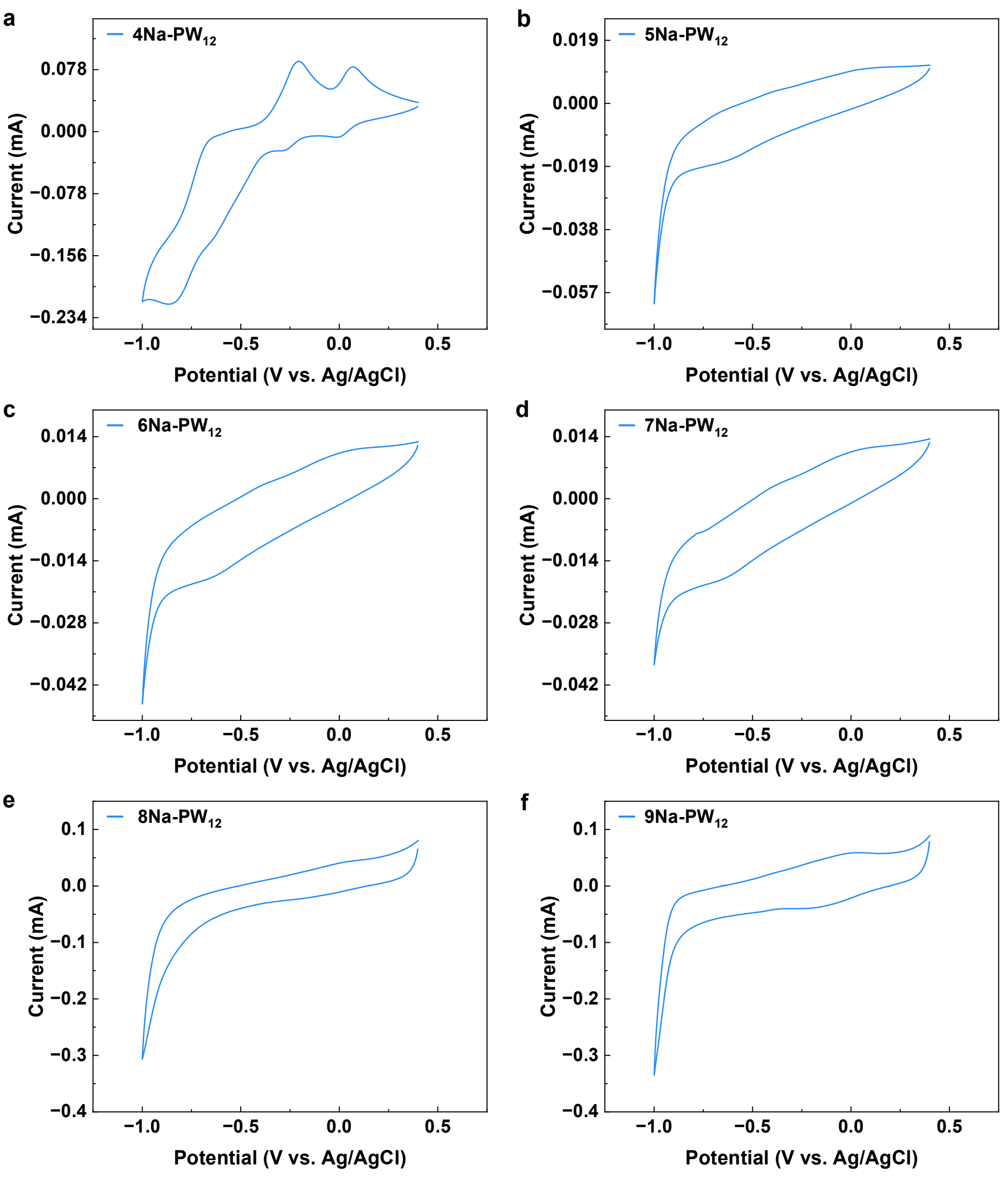

Supplementary Figure 3 | CV measurement. CV curves of 0.1 mol l^{-1} nNa-PW_{12} (n=4, 5, 6, 7, 8, 9) solutions at the scan rate of 50 mV s^{-1}. GC electrode, Pt electrode and Ag/AgCl (saturated KCl) electrode were used as working electrode, counter electrode and reference electrode respectively.

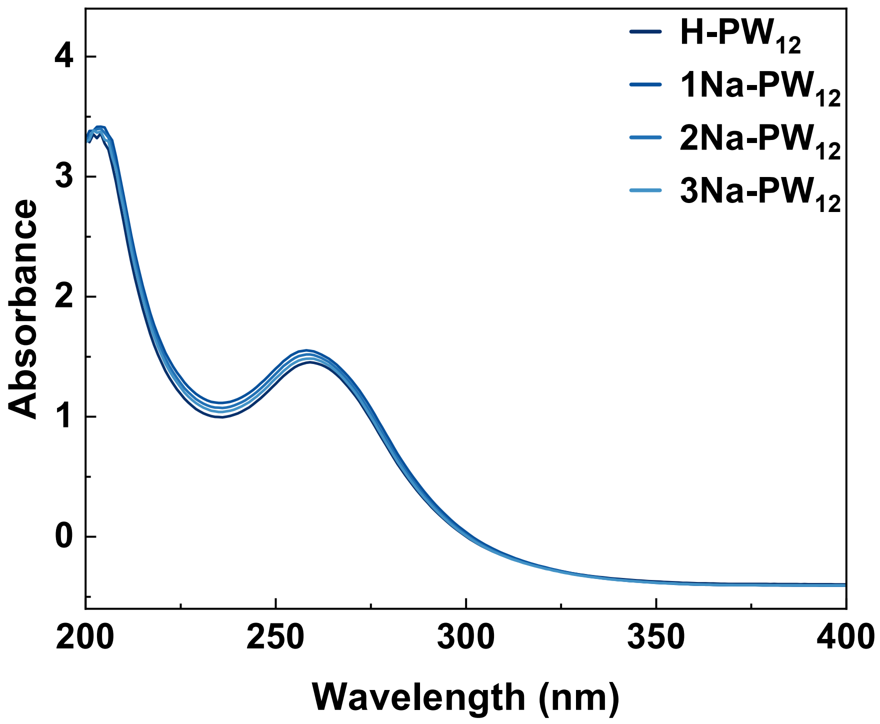

Supplementary Figure 4 | The UV-Vis spectrum of 0.33 mmol l^{-1} H-PW_{12}, 1Na, 2Na and 3Na-PW_{12} solutions.

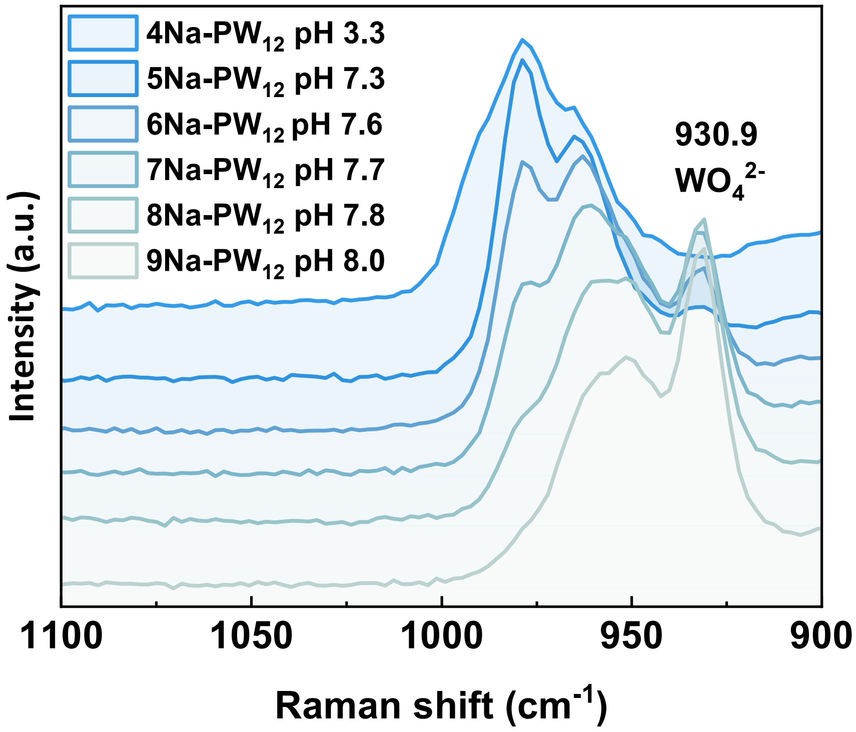

Supplementary Figure 5 | Raman spectrums of nNa-PW_{12}(n=4, 5, 6, 7, 8, 9) solutions. nNa-PW_{12} (n=4, 5, 6, 7, 8, 9) was prepared based on the mole ratio of NaOH and PW_{12}.

$$PW_{12} \underset{WO_4^{2-} + H^+}{\overset{(1)\ 6OH^-}{\rightleftharpoons}} PW_{11} \underset{WO_4^{2-} + H^+}{\overset{(2)\ 6OH^-}{\rightleftharpoons}} PW_9 \underset{WO_4^{2-} + H^+}{\overset{(3)\ 12OH^-}{\rightleftharpoons}} WO_4^{2-} + PO_4^{3-}$$

(1): $[PW_{12}O_{40}]^{3-} + OH^- \longrightarrow [PW_{11}O_{39}]^{7-} + WO_4^{2-} + H_2O$

(2): $3[PW_{11}O_{39}]^{7-} + 6OH^- \longrightarrow 2[PW_9O_{34}]^{9-} + 3WO_4^{2-} + 3H_2O$

(3): $[PW_9O_{34}]^{9-} + 12OH^- \longrightarrow 9WO_4^{2-} + PO_4^{3-} + 6H_2O$

Supplementary Figure 6 | The decomposition pathways of PW_{12} under alkaline condition.

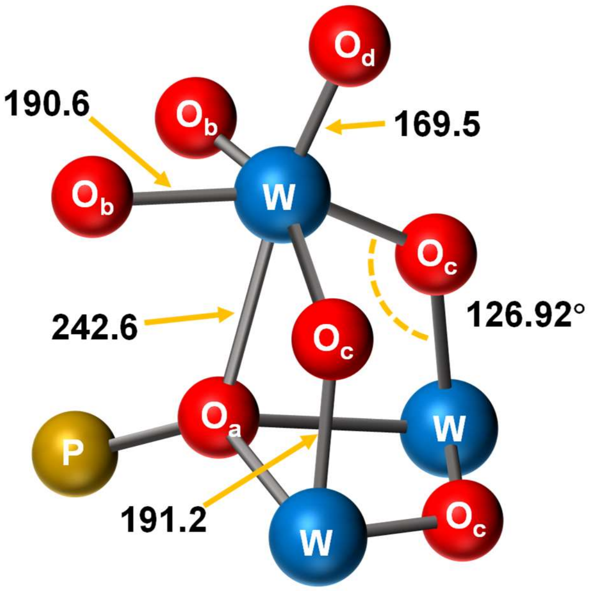

Supplementary Figure 7 | Structural illustration of 0e$^-$ PW_{12} subunit.

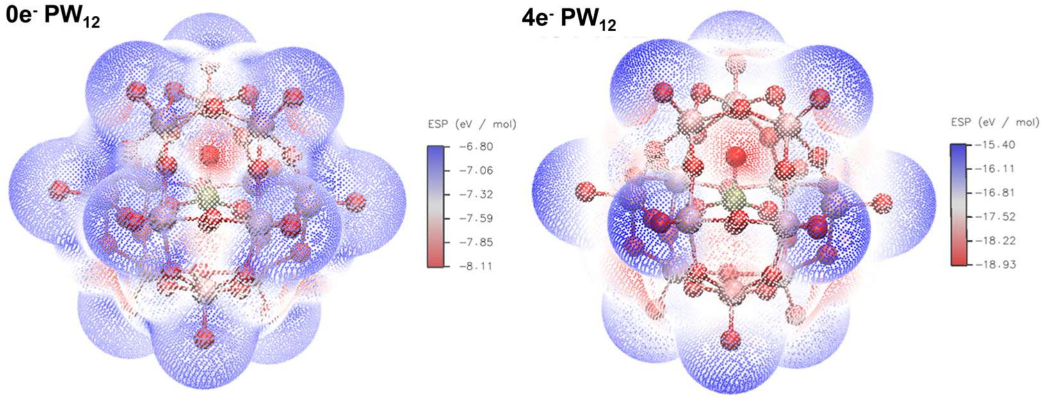

Supplementary Figure 8 | Electron static potential (ESP) distribution of 0e$^-$ PW_{12} and 4e$^-$ PW_{12}.

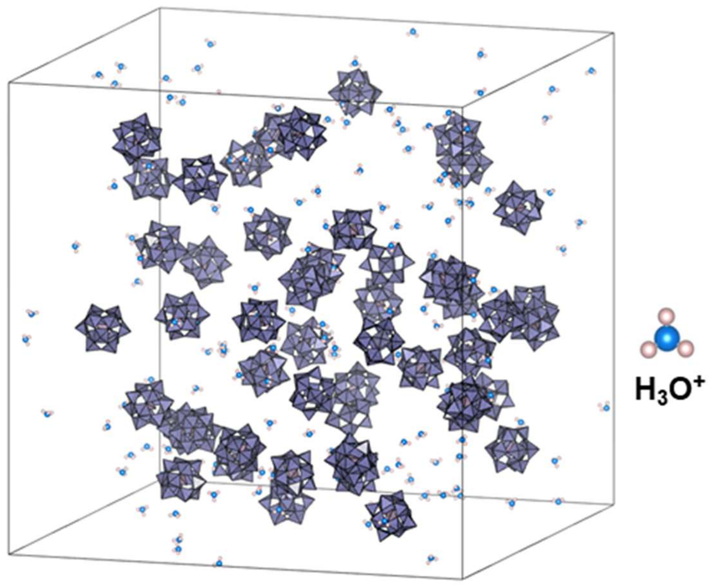

Supplementary Figure 9 | Snapshot of a representative 3D-periodic simulation box for 0e$^-$ PW_{12} MD simulations.

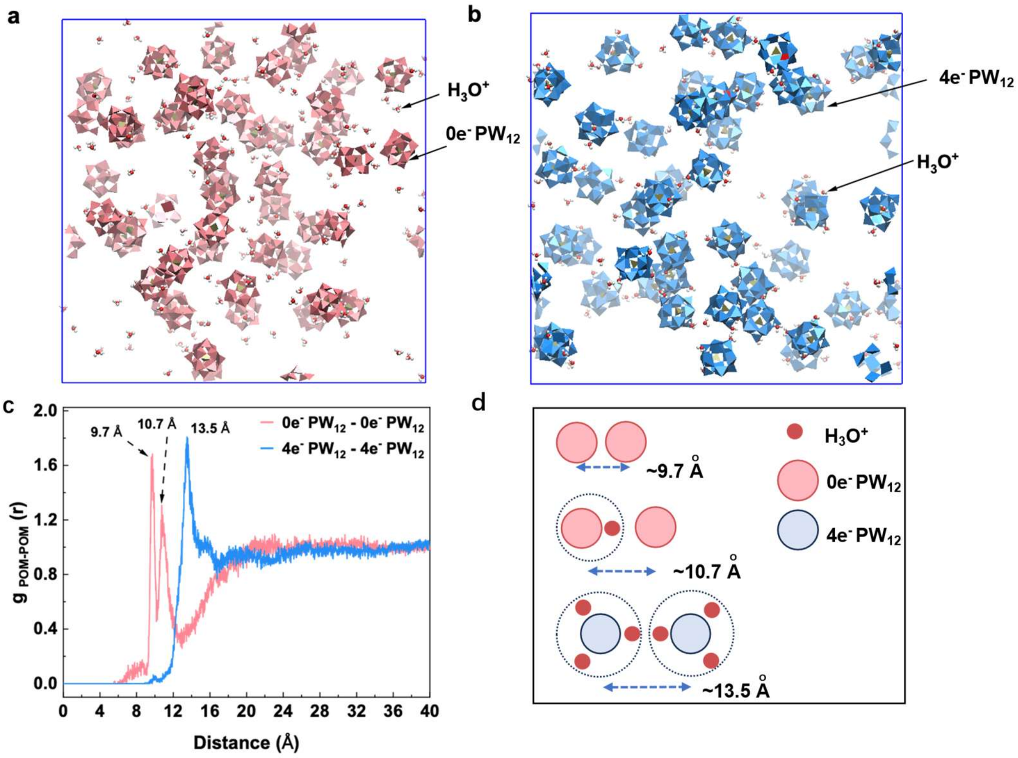

Supplementary Figure 10 | MD simulations. a. A snapshot of $0e^-$-PW_{12} simulation box. **b.** A snapshot of $4e^-$-PW_{12} simulation box. **c.** POM-POM radial distribution functions (RDFs) computed from classical MD simulations taking as reference the center of mass of each PW_{12}. **d.** The illustrations of the distance between two PW_{12} anions.

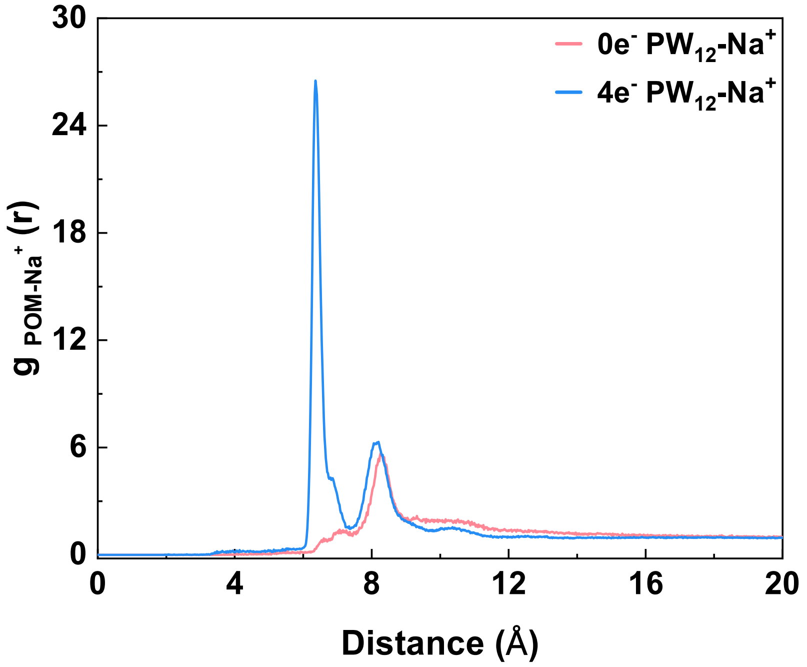

Supplementary Figure 11 | POM-Na^+ radial distribution functions (RDFs) calculated from classical MD simulations using the center of mass of each PW_{12} as reference.

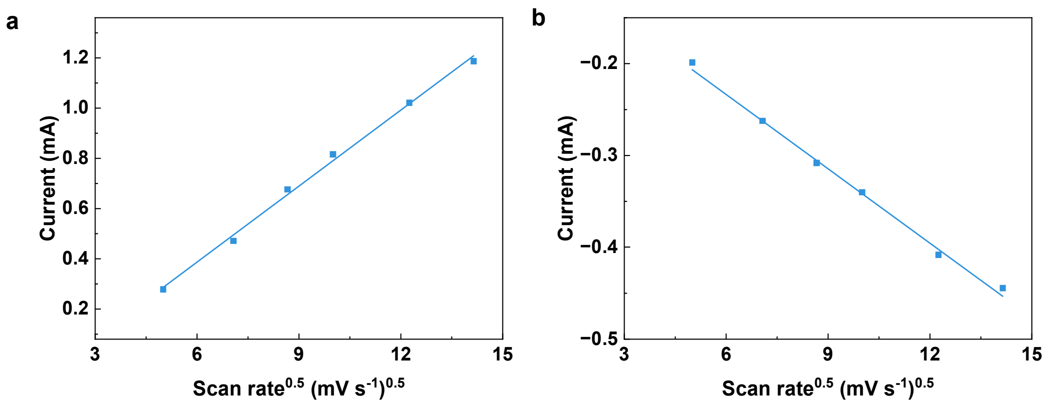

Supplementary Figure 12 | Randles-Sevcik equation. a, The first oxidation peak and **b,** the first reduction peak current density as a function of the square root of the scan rate obtained in Fig. 5a.

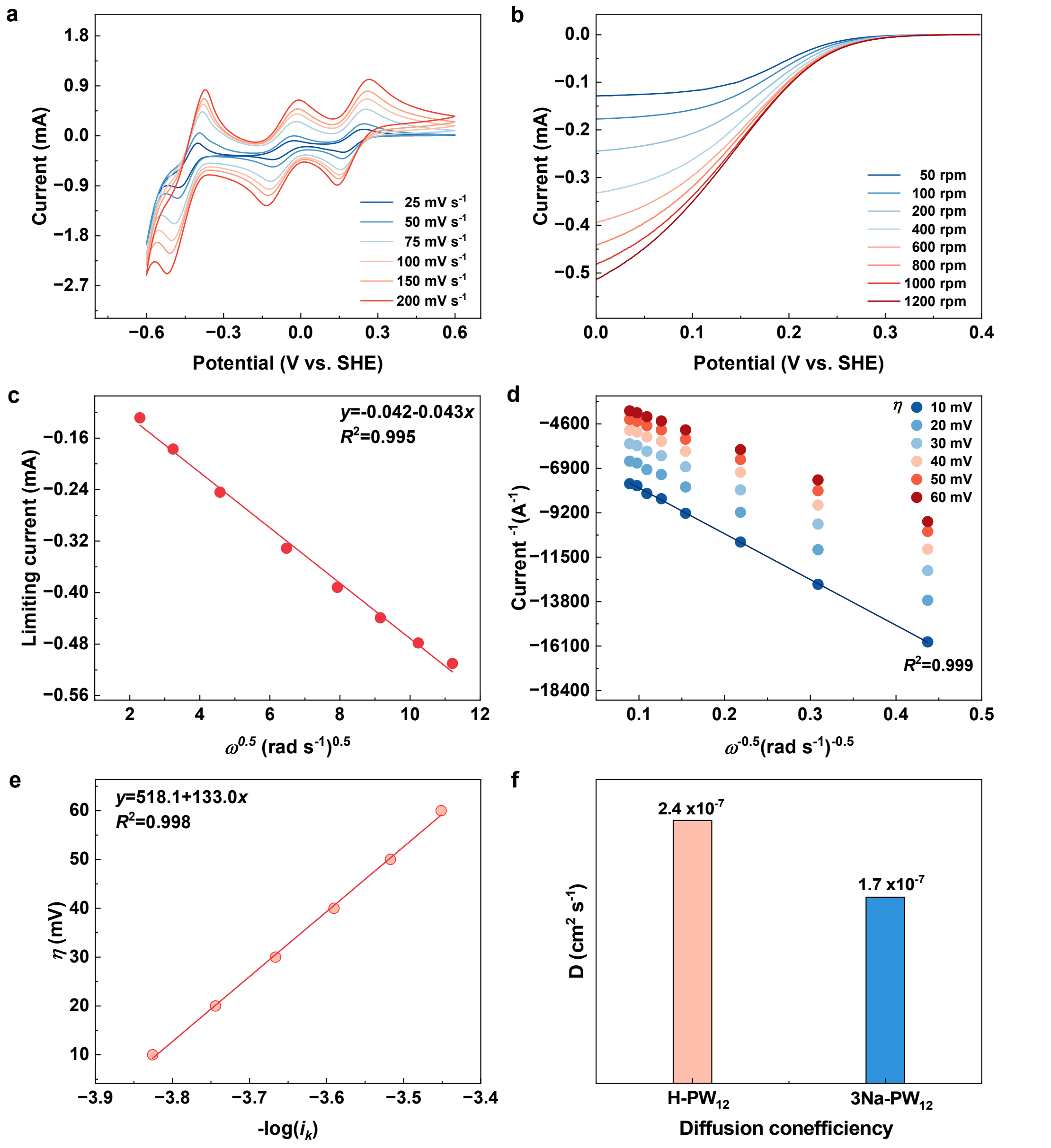

Supplementary Figure 13 | Electrochemical kinetics of H-PW₁₂. a) CV curves of 0. 1 mol l^{-1} H-PW_{12} with different scan rates. b) RDE tests with different rotation speeds, c) Levich plot, d) Koutecky–Levich plots and e) Butler–Volmer plot of 0.01 mol l^{-1} H-PW_{12}, f) diffusion coefficients of 0.01 mol l^{-1} H-PW_{12} and 3Na-PW_{12}

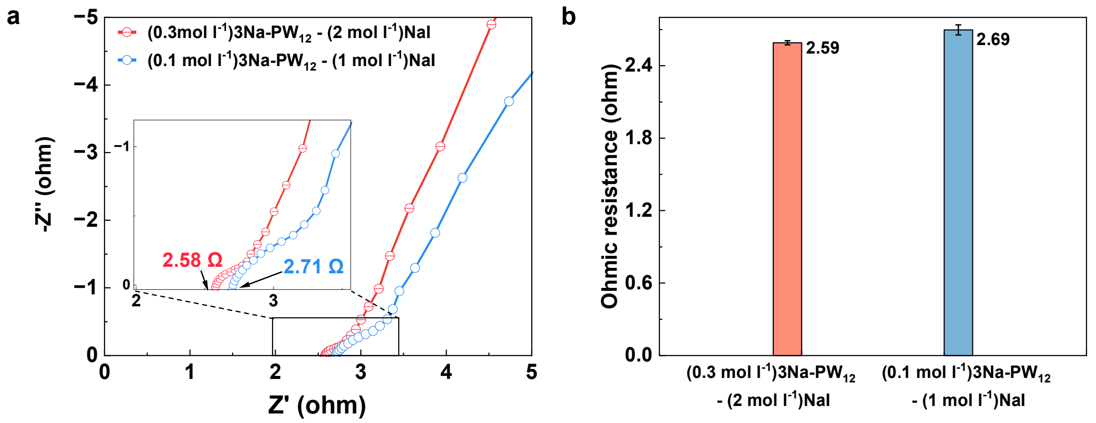

Supplementary Figure 14 | The ohmic resistance measurements of the flow battery. a) The typical Nyquist plots of electrochemical impedance spectroscopy (EIS) result of (0.1 mol l^{-1}) 3Na-PW_{12} - (1 mol l^{-1}) NaI and (0.3 mol l^{-1}) 3Na-PW_{12} - (2 mol l^{-1}) NaI redox flow batteries. The magnified plots are interceptions of the high-frequency parts and the x-axis. b) The summary of ohmic resistance values of the flow battery (electrode area: 16 cm^2, flow rate: 90 ml min^{-1}, room temperature).

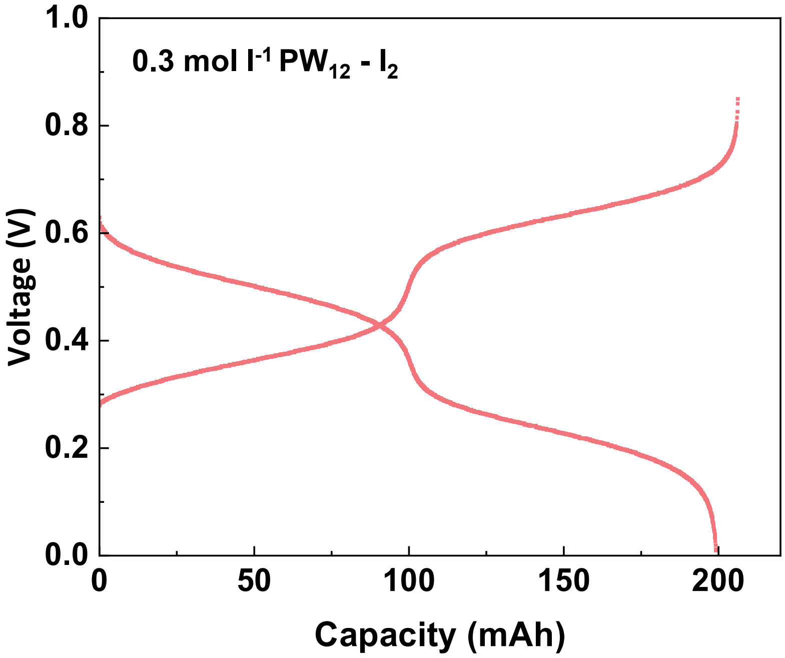

Supplementary Figure 15 | GCD curve of 0.3 mol l^{-1} H-PW_{12}-I_2 at current density of 25 mA cm^{-2}

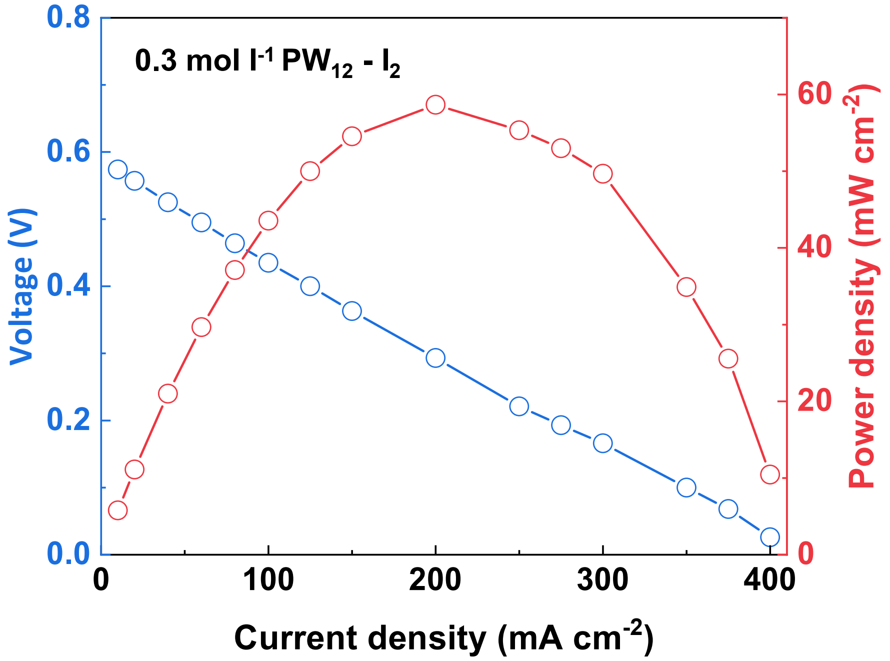

Supplementary Figure 16 | Discharge polarization curve of 0.3 mol l^{-1} H-PW_{12}-I_2.

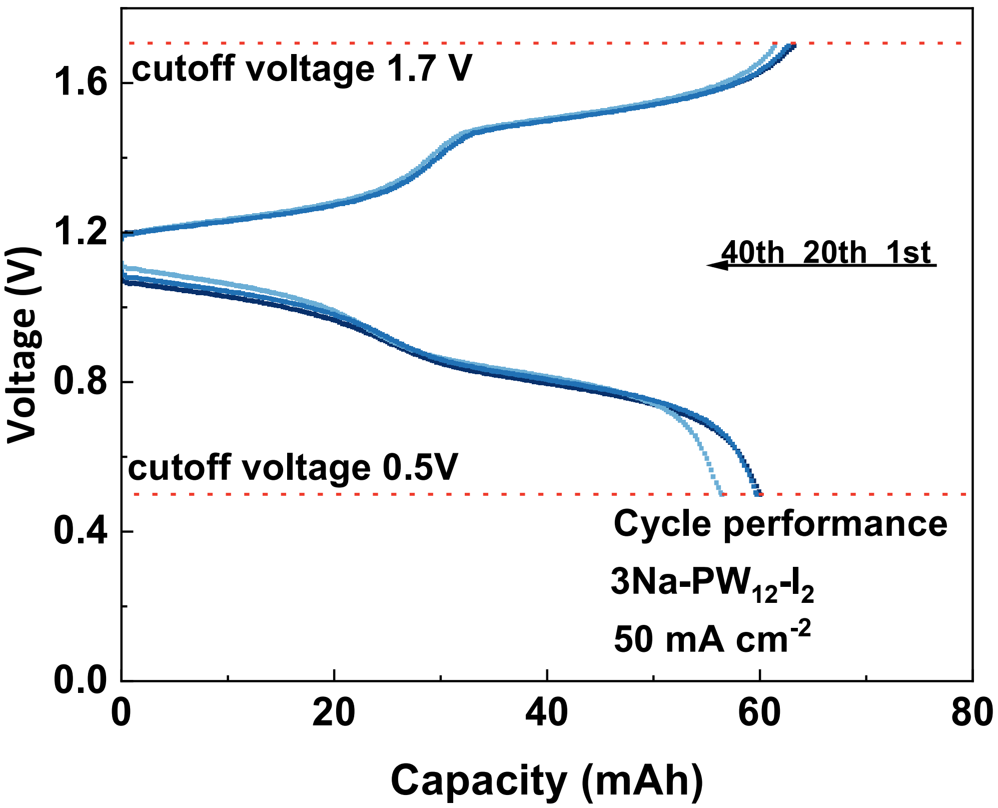

Supplementary Figure 17 | The GCD curves of cycle performance (0.1 mol l^{-1} 3Na-PW_{12}-I_2) at current density of 50 mA cm^{-2}.

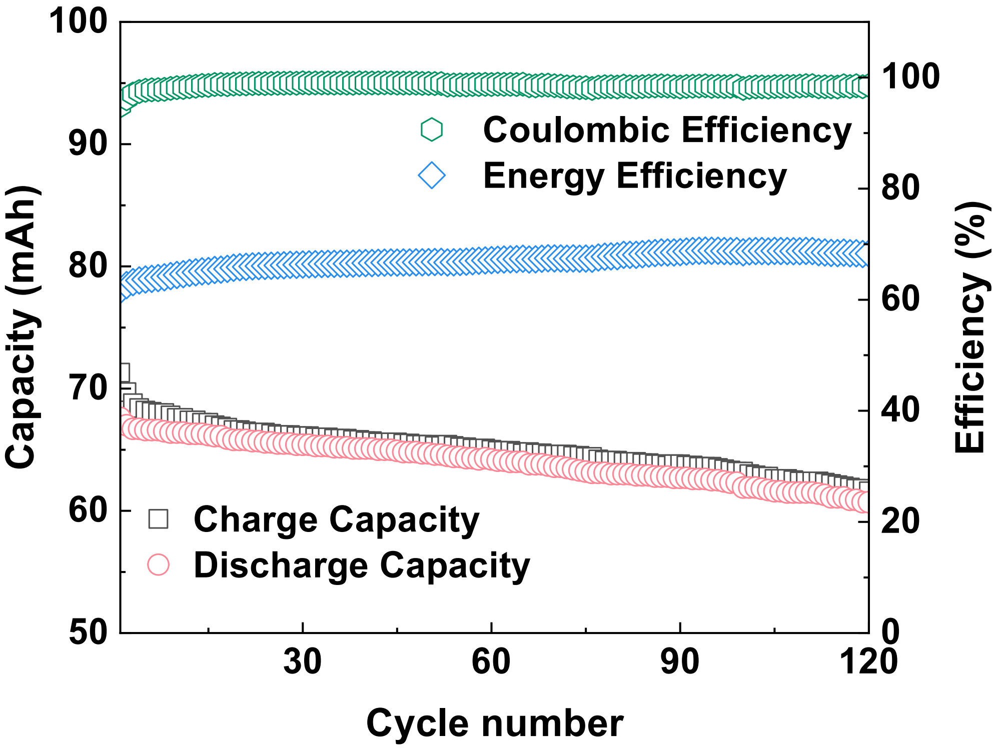

Supplementary Figure 18 | The reproducibility of the aqueous flow battery. 120 cycles GCD performances of 3Na-PW_{12}-NaI redox flow battery (0.1 mol l^{-1} 3Na-PW_{12}, and 1 mol l^{-1} NaI, constant current density: 50 mA cm^{-2}, flow rate: 90 ml min^{-1}, room temperature, electrode area: 16 cm^2).

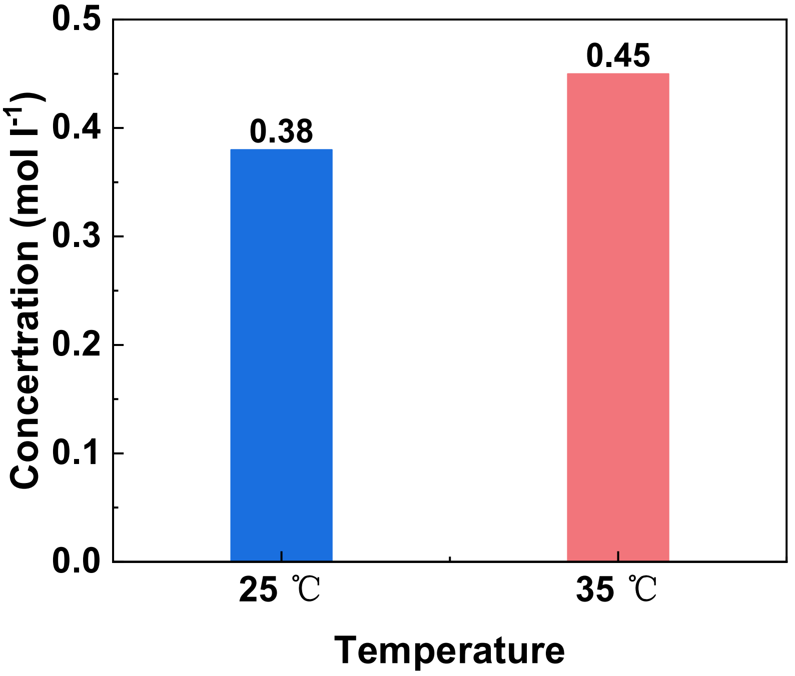

Supplementary Figure 19 | The solubility of the active material 3Na-PW_{12} at 25 and 35 ºC.

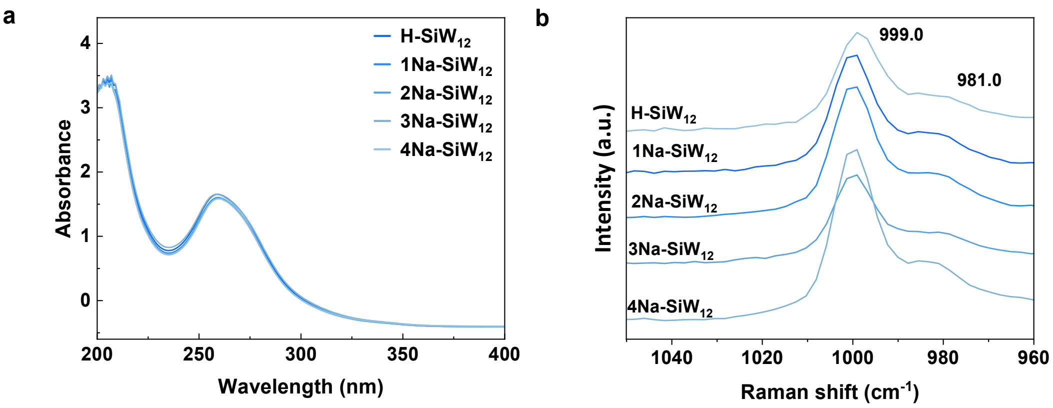

Supplementary Figure 20 | UV and Raman characterizations of SiW_{12}. a, UV-Vis spectra of 0.33 mmol l^{-1} H, 1Na, 2Na, 3Na and 4Na-SiW_{12}. **b,** Raman spectra of H, 1Na, 2Na, 3Na and 4Na-SiW_{12}。

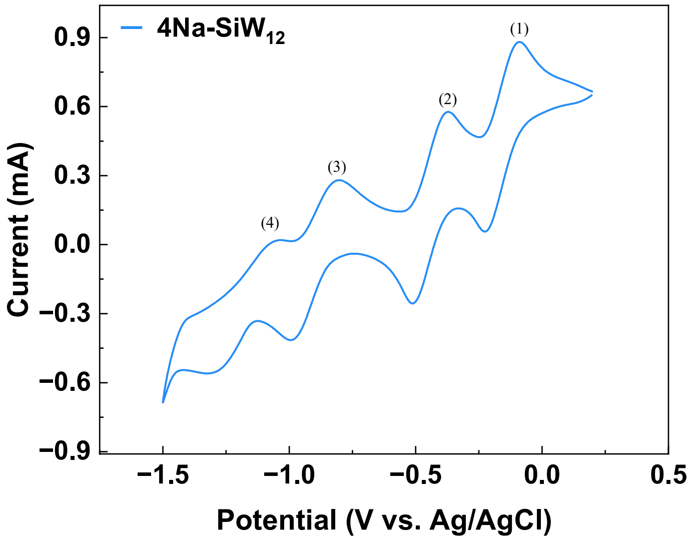

Supplementary Figure 21 | CV curve of 0.1 mol l^{-1} reduced 4Na-SiW_{12} (0.1 mol l^{-1}) at the scan rate of 50 mV s^{-1}. GC electrode, Pt electrode and Ag/AgCl (saturated KCl) electrode were used as working electrode, counter electrode and reference electrode respectively. The CV curve of 4-Na SiW_{12} shows 4 characteristic peaks, which can be ascribed to 4 single electron transfer reactions under the neutral concentration: (1) $SiW_{12}^{4-} + e \rightarrow SiW_{12}^{5-}$ E_1 = -0.15 V (vs Ag/AgCl); (2) $SiW_{12}^{5-} + e \rightarrow SiW_{12}^{6-}$ E_2= -0.45 V (vs Ag/AgCl); (3) $SiW_{12}^{6-} + e \rightarrow SiW_{12}^{7-}$ E_3 = -0.90 V (vs Ag/AgCl); (4) $SiW_{12}^{7-} + e \rightarrow SiW_{12}^{8-}$ E_4 = -1.19 V (vs Ag/AgCl).[1]

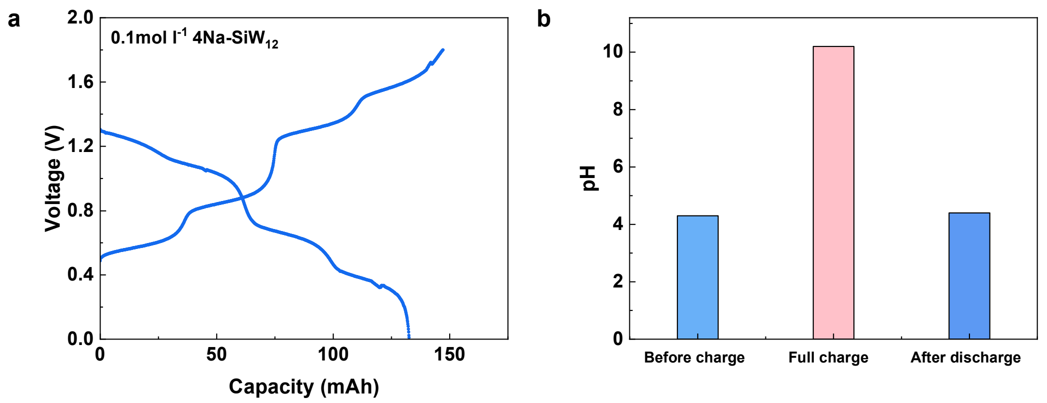

Supplementary Figure 22 | Electrochemical performance of 4Na-SiW$_{12}$ Flow battery. a, GCD curve of 0.1 mol l^{-1} 4Na-SiW$_{12}$- I_2 at 25 mA cm^{-2}. b) pH changes of 4Na-SiW$_{12}$ in the GCD process.

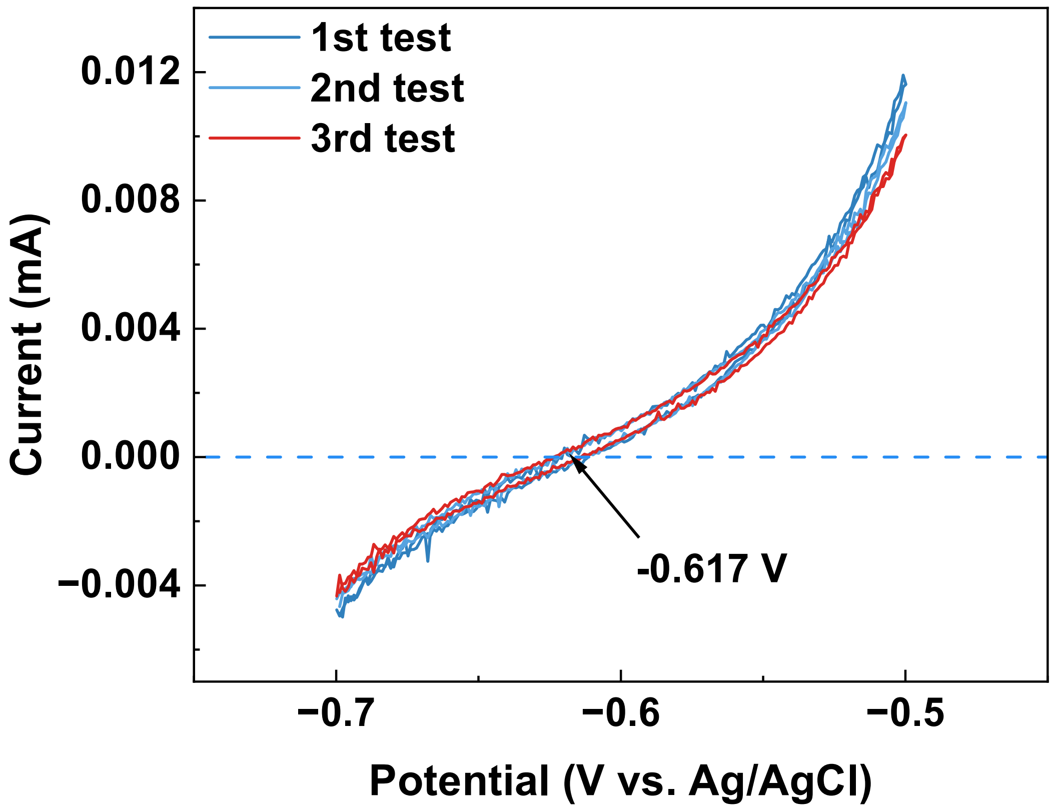

Supplementary Figure 23 | Calibration curves of Ag/AgCl electrode in saturated KCl solution at room temperature (pH=6.90). The measured reversible hydrogen evolution potential is -0.617 V Vs Ag/AgCl electrode. According to the calculation equation $E_{\mathrm{measured}} = E_{\mathrm{RHE}} - E_{\mathrm{Ag/AgCl}}$, the potential of Ag/AgCl electrode can be calculated as follows: $E_{\mathrm{Ag/AgCl}}$ = -0.4085+0.6170 = 0.2085 V vs. SHE, which is very close to the standard Ag/AgCl electrode potential of 0.199 V vs. SHE in saturated KCl solution at 25 °C. Based on the calibration of Ag/AgCl electrode, 0.2085 was used as actual potential of Ag/AgCl electrode in this work.

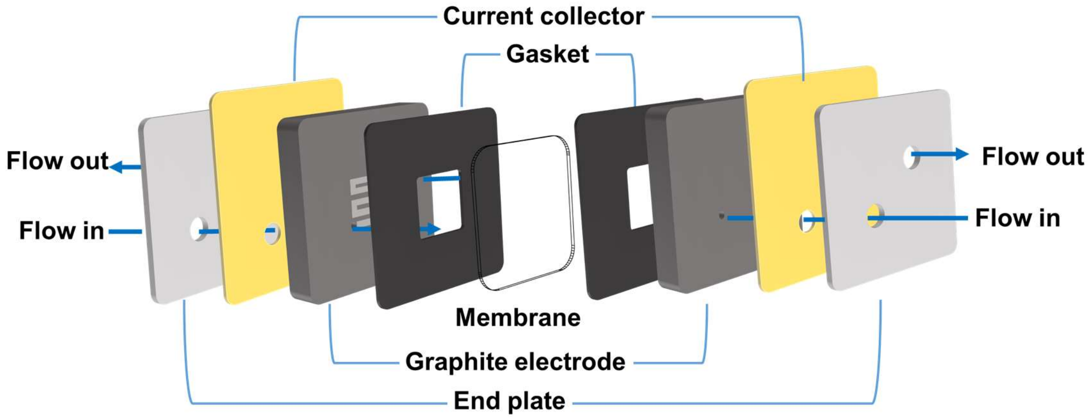

Supplementary Figure 24 | The structure of the flow battery. The flow battery used in the experiment was homemade with a computer numerical control (CNC) mill. The battery was assembled with proton exchange membrane, gasket, graphite electrode (10 mm thick), current collector (Cu, 1.5 mm thick) and end plate (PP, 5 mm thick). Graphite felt electrode (2 mm thick) was used to increase the contact area between electrolyte and graphite electrode. The 'S' shape flow field with 1 mm depth and the area of 9 cm^2 and 16 cm^2 respectively were prepared. Nafion® 117 was used as the membrane in the experiment without pretreatment.

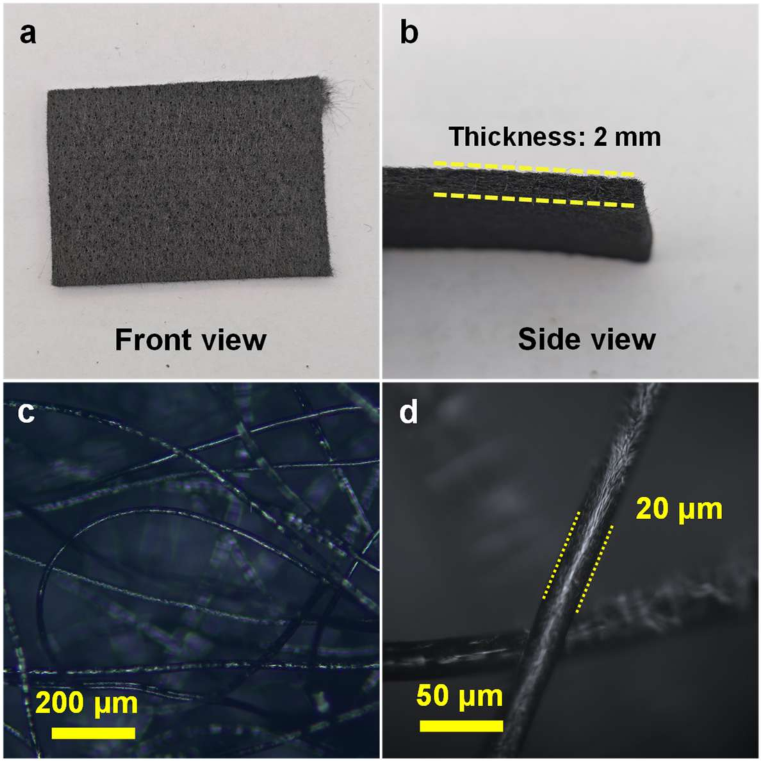

Supplementary Figure 25 | The digital photographs of the employed commercial graphite felt electrode in the flow battery: (a) The front view, (b) The side view. Optical micrographs of graphite felts (c) magnification of 100x, (d) magnification of 400x.

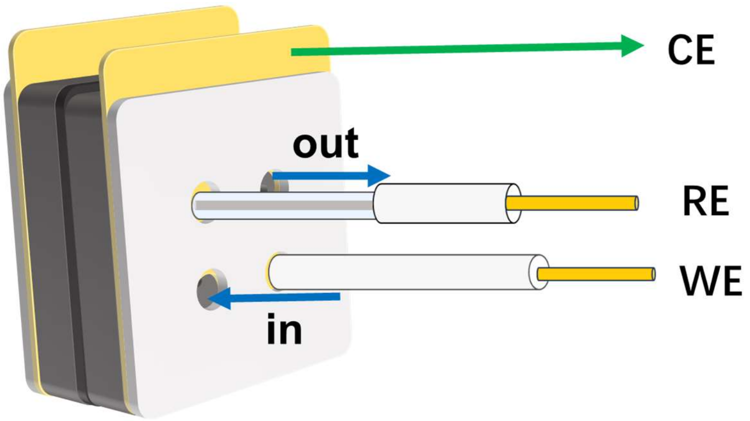

Supplementary Figure 26 | The Structure of flow battery for in-situ CV and electrode potential measurements. The Ag/AgCl (saturated KCl) electrode and GC electrode were used as reference electrode and working electrode.

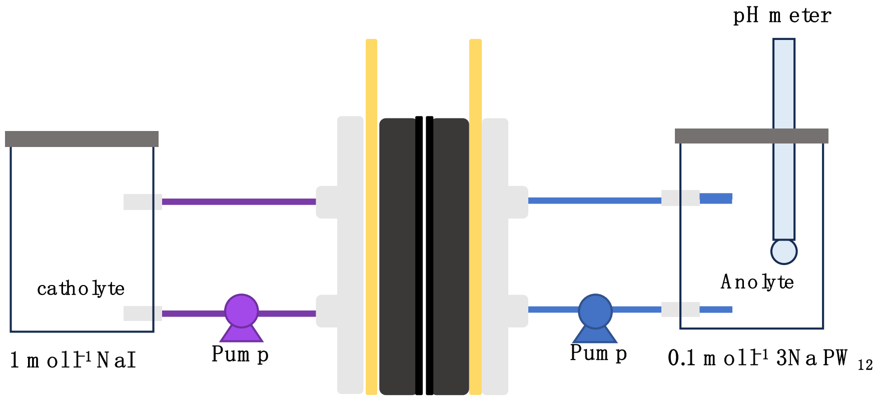

Supplementary Figure 27 | The illustration of in-situ pH measurement. The test was carried out in 0.1 mol l^{-1} 3Na-PW_{12} solution at current density of 25 mA cm^{-2} with 1 mol l^{-1} NaI (pH 1.35) used as catholyte.

Supplementary Table 1 |Potentials of redox pairs.

Redox pair	Potential (V vs. SHE)	Condition	Reference
$PW_{12}^{4-}/PW_{12}^{5-}$	-0.07	pH<1	[2]
$SiW_{12}^{5-}/SiW_{12}^{6-}$	-0.25	pH<1	[3]
V^{2+}/V^{3+}	-0.27	pH<1	[4]
$P_2W_{18}^{10}/P_2W_{18}^{12-}$	-0.31	pH<1	[4]
Fe/Fe^{2+}	-0.44	pH=7	[5]
$BW_{12}^{6-}/BW_{12}^{7-}$	-0.46	pH 1-8	[6]
$CoW_{12}^{8-}CoW_{12}^{10-}$	-0.47	pH=4	[7]
Zn/Zn^{2+}	-0.76	pH=7	[8]
**$PW_{12}^{5-}/PW_{12}^{6-}$**	**-0.51**	**pH=6.1**	**This work**
**$SiW_{12}^{6-}/SiW_{12}^{7-}$**	**-0.70**	**pH=8.7**	**This work**
**$PW_{12}^{6-}/PW_{12}^{7-}$**	**-0.78**	**pH=11**	**This work**
**$SiW_{12}^{7-}/SiW_{12}^{8-}$**	**-0.99**	**pH=8**	**This work**
**$PW_{12}^{7-}/PW_{12}^{8-}$**	**-1.1 V**	**pH=10.4**	**This Work**

Supplementary Table 2 | Electron numbers utilized by different POMs redox pairs in flow battery.

POM	Electron numbers	Atomic utilization(%)	Reference
PW_{12}	2	16.7	[2]
BW_{12}	2	16.7	[6]
SiW_{12}	2	16.7	[3]
PV_{14}	4	28.6	[3]
P_2W_{18}	18	100	[9]
CoW_{12}	4	33.3	[7]
SiW_9V_3	3	25.0	[10]
**3Na PW_{12}**	5	41.7	**This work**
**4Na SiW_{12}**	4	33.3	**This work**

Supplementary Table 3 | Rate performances of 0.1 mol l^{-1} 3Na-PW_{12} : 1 mol l^{-1} NaI flow battery

**Current density ($mA\ cm^{-2}$)**	**Specific Capacity ($Ah\ L^{-1}$)**	**Columbic efficiency (%)**
20	8.3	95.65
30	7.8	97.15
40	7.2	97.76
50	6.4	98.22
60	5.5	98.65
100	2.3	98

(Electrolyte: 15 ml 1 mol l^{-1} NaI; 15ml 0.1 mol l^{-1} 3Na-PW_{12})

Supplementary Table 4 |The viscosity of the electrolytes.

	**kinematic viscosity (cm^2 s^{-1})**
0.01 mol l^{-1} $H\text{-}PW_{12}$	0.00879
0.01 mol l^{-1} $3Na\text{-}PW_{12}$	0.00904

Supplementary References

1. Guo, S.-X., Mariotti, A. W. A., Schlipf, C., Bond, A. M. & Wedd, A. G. A Systematic approach to the simulation of the voltammetric reduction of $[\alpha\text{-}SiW_{12}O_{40}]^{4-}$ in buffered aqueous electrolyte media and acetonitrile. *J. Electroanal. Chem.* **591**, 7–18 (2006).
2. Feng, T. *et al.* A redox flow battery with high capacity retention using 12-phosphotungstic acid/iodine mixed solution as electrolytes. *J. Power Sources* **436**, 226831 (2019).
3. Friedl, J. *et al.* Asymmetric polyoxometalate electrolytes for advanced redox flow batteries. *Energy Environ. Sci.* **11**, 3010–3018 (2018).
4. Ai, F. *et al.* Heteropoly acid negolytes for high-power-density aqueous redox flow batteries at low temperatures. *Nat. Energy* **7**, 417–426 (2022).
5. Song, Y. *et al.* Simultaneous regulation of solvation shell and oriented deposition toward a highly reversible fe anode for all-iron flow batteries. *Small* **18**, 2204356.
6. Yang, L. *et al.* POM anolyte for all-anion redox flow batteries with high capacity retention and coulombic efficiency at mild pH. *Adv. Mater.* **34**, 2107425 (2022).
7. Yang, L. *et al.* Tuning the inner- and outer-sphere electron transfer behavior of aqueous $\{CoW_{12}\}$ polyoxometalate clusters for redox flow batteries exceeding 1.5V.

Energy Storage Mater. **65**, 103149 (2024).

8. Xie, C., Liu, Y., Lu, W., Zhang, H. & Li, X. Highly stable zinc–iodine single flow batteries with super high energy density for stationary energy storage. *Energy Environ. Sci.* **12**, 1834–1839 (2019).

9. Chen, J.-J., Symes, M. D. & Cronin, L. Highly reduced and protonated aqueous solutions of $[P_2W_{18}O_{62}]^{6-}$ for on-demand hydrogen generation and energy storage. *Nat. Chem.* **10**, 1042–1047 (2018).

10. Pratt, H. D., Hudak, N. S., Fang, X. & Anderson, T. M. A polyoxometalate flow battery. *J. Power Sources* **236**, 259–264 (2013).
